# Epidemiological Review of accidents and events in Sabzevar province during 2005-2013 

**Published:** 2019-08

**Authors:** Rahim Akrami, Seyed Mohsen Mehri, Maryam Masudifar

**Affiliations:** ^*a*^Deputy of Research and Technology, Sabzevar University of Medical Sciences, Sabzevar, Iran.; ^*b*^Deputy of Health, Sabzevar University of Medical Sciences, Sabzevar, Iran.

## Abstract

**Background:**

The most important ways to deal with events, a prevention is needed to recognize the epidemiology of incidents and identification Factors and Variables Epidemiologically, with attention To Area Geographic and Grade scale Time. Since the city of Sabzevar in Khorasan Razavi province is the third most populous city, Epidemiologic review the importance of incidents in this city. The purpose of this study was to evaluate the epidemiological situation of accidents and events in Sabzevar city in the year 1384 to 1391.

**Methods:**

This cross-sectional descriptive study was conducted in which 68346 patients were injured From March 1384 (21 March 2005) to 30 Esfand 1391 (20 March 2013) accident and were admitted to a hospital in the city of Sabzevar Collected and analyzed. Data collection was done using hospital registration form. Finally the data is provided by the software STATA Version 11 was analyzed.

**Results:**

All events recorded in the city of Sabzevar during 8 years, were about 68,346. According to gender the majority of people were men (69.82 %) and the least were women (30.18. Average age of these people 27.20 ± 4.17 and in the range of under 1 year to 99 years. Most of the accidents, the age group 15-24 years (33.39%) and 25- 34 years (20.70%) reported the lowest the following year (2. 36%), respectively. Among the types of accidents, motorcycle accidents by 25.88% cases, the most has taken over. Motorcycle crashes (30.49%), the most common type of injury among men And the other most cases among female victims (21.7%, respectively). Urban areas are the most frequent events with 66.66% of cases. The highest incidence is related to the location of street (36%) and home (30.94%) and the lowest number of places are sports (1.16%) and schools (1.27%). In terms of the frequency of events by season, spring was the most prevalent with 30.92 % and summer with 29.42% of cases. Most of the injured (95.56%) were treated and 0.23 % respectively and 3.75% of the cases were fatal and disability.

**Conclusions:**

It is necessary to provide public education about the common accidents in these places and ways to prevent them through the mass media, while improving the unsafe conditions and making the streets safer. More attention needs to be paid to preventive measures and to strengthening the provision of health services with the aim of rapidly recovering and returning to work.

**Keywords:**

Accidents and events, Sabzevar, Hospital, Disability, Death

